# Classification of Different Motor Imagery Tasks with the Same Limb Using Electroencephalographic Signals

**DOI:** 10.3390/s25175291

**Published:** 2025-08-26

**Authors:** Eric Kauati-Saito, André da Silva Pereira, Ana Paula Fontana, Antonio Mauricio Ferreira Leite Miranda de Sá, Juliana Guimarães Martins Soares, Carlos Julio Tierra-Criollo

**Affiliations:** 1 Laboratory of Medical Signal and Images Processing, Biomedical Engineering Program, Alberto Luiz Coimbra Institute for Graduate Studies and Research in Engineering (COPPE), Federal University of Rio de Janeiro (UFRJ), Rio de Janeiro 21941-901, Brazil; 2 Laboratory of Cognitive Physiology, Biophysics Institute Carlos Chagas Filho, Federal University of Rio de Janeiro (UFRJ), Rio de Janeiro 21941-902, Brazil; jmsoares@biof.ufrj.br; 3 Rehabilitation Sciences Program, School of Physiotherapy, Neurology Institute Deolindo Couto, Federal University of Rio de Janeiro (UFRJ), Rio de Janeiro 22290-140, Brazil

**Keywords:** electroencephalography, brain–computer interface, feature extraction, classification

## Abstract

Stroke is a neurological condition that often results in long-term motor deficits. Given the high prevalence of motor impairments worldwide, there is a critical need to explore innovative neurorehabilitation strategies that aim to enhance the quality of life of patients. One promising approach involves brain–computer interface (BCI) systems controlled by electroencephalographic (EEG) signals elicited when a subject performs motor imagery (MI), which is the mental simulation of movement without actual execution. Such systems have shown potential for facilitating motor recovery by promoting neuroplastic mechanisms. Controlling BCI systems based on MI-EEG signals involves the following sequential stages: recording the raw signal, preprocessing, feature extraction and selection, and classification. Each of these stages can be executed using several techniques and numerous parameter combinations. In this study, we searched for the combination of feature extraction technique, time window, frequency range, and classifier that could provide the best classification accuracy for the BCI Competition 2008 IV 2a benchmark dataset (BCI-C), characterized by EEG-MI data of different limbs (four classes, of which three were used in this work), and the NeuroSCP EEG-MI dataset, a custom experimental protocol developed in our laboratory, consisting of EEG recordings of different movements with the same limb (three classes—right dominant arm). The mean classification accuracy for BCI-C was 76%. When the subjects were evaluated individually, the best-case classification accuracy was 94% and the worst case was 54%. For the NeuroSCP dataset, the average classification result was 53%. The individual subject’s evaluation best-case was 71% and the worst case was 35%, which is close to the chance level (33%). These results indicate that techniques commonly applied to classify different limb MI based on EEG features cannot perform well when classifying different MI tasks with the same limb. Therefore, we propose other techniques, such as EEG functional connectivity, as a feature that could be tested in future works to classify different MI tasks of the same limb.

## 1. Introduction

A stroke is a sudden and irreversible clinical event that leads to neural cell death by interruption of the oxygen supply [1]. Stroke can affect several brain regions, including the basal ganglia, brainstem, cerebellum, and subarachnoid space. It affects around 14 million people every year. Of this number, around one-third of these individuals suffer from permanent motor disabilities, making it the leading cause of motor deficits worldwide [2,3]. In this scenario, marked by a high number of stroke survivors with motor deficits, motor imagery (MI) has emerged as an auxiliary physiotherapy approach, with positive results reported in the scientific literature [4,5,6,7,8]. MI can be defined as a dynamic state in which the neural representation of a specific action is reactivated within the working memory without any overt motor action. Both MI and the execution of a movement are governed by the same principles of central motor control [9,10].

IM-based therapy is capable of stimulating reorganization of neuronal motor networks. There is significant evidence for the role of MI in rehabilitation as it promotes cortical reorganization and increases the resting-state functional connectivity between the ipsilesional M1 and ipsilesional precentral and postcentral gyri, middle cingulate gyrus, and supramarginal gyrus [11]. MI also promotes the activation of several brain regions, similar to motor execution, such as the supplementary motor areas, dorsal and ventral premotor cortex, cingulate gyrus, putamen, parietal lobes, basal ganglia, and cerebellum [12]. Furthermore, additional activation in the frontal brain areas is associated with IM [13].

The brain signals elicited when a subject performs the MI of a specific movement can be used to control external devices through a paradigm known as brain–computer interfaces (BCI). These systems convert the user’s electrophysiological activity into output signals capable of managing applications such as orthoses or wheelchairs [14]. BCI-IM systems allow an increasing number of people suffering from diseases that impair movement control to improve both their interaction with the environment and their rehabilitation process. The control of BCI systems based on the user’s MI can improve post-stroke rehabilitation outcomes when compared to robotic therapy not controlled by MI [15]. Therefore, the coupling of BCI-MI can improve the quality of life by enhancing upper-limb motor recovery and promoting autonomy in users [16].

EEG is a non-invasive technique that allows for high temporal resolution recordings of brain activity while subjects perform MI. Controlling a brain–computer interface (BCI) using EEG signals involves a series of sequential stages. First, EEG activity is recorded from the scalp. The raw signals are then preprocessed to remove artifacts and enhance signal quality. Following this, relevant features are extracted and selected to capture the neural patterns associated with the user’s intent. These features are subsequently classified and translated into digital commands that reflect the user’s intended action. Finally, these commands are used to control external devices, enabling real-time interaction between the brain and the environment [17].

One of the main challenges when applying BCI-MI systems is achieving decent decoding accuracy from EEG signals, given the significant inter-session and intersubject variability [18]. For this reason, many different feature extraction and feature selection techniques have been applied in the literature, such as Common Spatial Patterns (CSP) [18,19,20], Filter Bank Common Spatial Patterns (FB-CSP) [21], Riemannian Geometry-Based Approaches (RGBA) [22], Deep Learning-Based Features [23], Independent Component Analysis [24], Autoregressive Modeling [25], and Wavelet Transform [26] and many others.

Another challenge faced by BCI-MI systems is the classification of different tasks performed with the same limb using features extracted from ongoing EEG activity. This capability is essential for enabling greater dimensional control of the BCI system [27] and increases the likelihood of developing an intuitive and operable rehabilitation device [28]. Accurate classification of same-limb tasks is beneficial for applications such as neuroprostheses that require fine control and closed-loop BCI systems controlling Functional Electrical Stimulation (FES), where electrical stimulation can be selectively delivered to flexor or extensor muscles based on the classification of the user’s motor intention.

Table 1 presents a comparative overview of recent studies that focused on the classification of different MI tasks performed with the same limb. Most of the listed works employ traditional feature extraction methods such as CSP, FB-CSP, or correlation-based features, as well as deep learning approaches, including convolutional neural network-based architectures (CNN). The number of classes varies across studies, with some addressing binary classification (flexion vs. extension) [27], while others explore more complex multiclass settings involving three or more tasks [29,30,31,32]. Reported accuracies range from 22.5% in a six-class setup [32] to over 90% in studies with smaller subject pools and optimized task distinctions [31].

In this study, we compare the performance of three different feature extraction techniques using EEG data from two sources: the BCI Competition IV 2a benchmark (BCI-C dataset), which includes MI of different limbs [34], and an original experimental protocol developed in our laboratory (NeuroSCP dataset), which comprises EEG recordings of different MI tasks involving the same limb. This work focuses on the underexplored challenge of classifying different motor imagery tasks involving the same limb, the use of both benchmark and custom datasets, and a systematic comparison of feature extraction techniques in different time windows. This approach provides practical insights for designing BCI systems with higher-dimensional control, which is particularly relevant for neurorehabilitation and neuroprosthetic applications.

The primary objective is to assess whether the classification accuracy of MI tasks involving the same limb can be significantly enhanced through the choice of feature extraction method. This question is particularly relevant because MI tasks involving the same limb tend to produce more similar neural patterns compared to tasks involving different limbs, making them inherently more challenging to discriminate using EEG signals.

## 2. Materials and Methods

### 2.1. BCI Competition Dataset

The BCI Competition 2008 IV 2a dataset consisted of EEG data from 9 subjects [34]. The cue-based BCI paradigm applied consisted of four different motor imagery tasks: IM of left-hand or right-hand movement, feet, and tongue. Each subject performed a total of 576 trials (144 trials for each class). The EEG was recorded with 22 channels using the following 10-20 electrode positions: Fz, FC3, FC1, FCz, FC2, FC4, C5, C3, C1, Cz, C2, C4, C6, CP3, CP1, CPz, CP2, CP4, P1, Pz, P2, and Oz. The recordings were made at a 250 Hz sampling rate, using the left mastoid as the reference and the right mastoid as the ground. The signals were band-pass filtered between 0.5 Hz and 100 Hz and notch-filtered at 50 Hz to suppress line noise. In this work, only EEG signals related to the right and left hands and feet MI were used.

### 2.2. NeuroSCP Dataset

**Figure 1:**
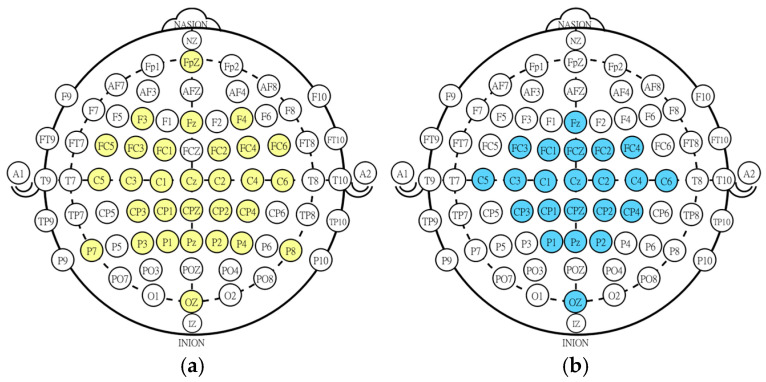
Electrode placement on the scalp according to the 10-10 system. (**a**) Yellow channels represent the channels used in the NeuroSCP dataset. (**b**) Blue channels represent the channels of the BCI Competition dataset.

The NeuroSCP dataset was recorded using a 40-channel data-acquisition system [35]. Of these, 32 were dedicated to EEG recordings using the following electrode positions: FPz, F3, Fz, F4, FC5, FC3, FC1, FC2, FC4, FC6, C5, C3, C1, Cz, C2, C4, C6, CP3, CP1, CPz, CP2, CP4, P7, P3, P1, Pz, P2, P4, P8, Oz, A1, and A2 (Figure 1). In addition, four electromyography (EMG) channels, one electrooculography (EOG) channel, and one accelerometer channel were also recorded. The EEG reference was placed at the Cz electrode, and the ground electrode was positioned on the participant’s forehead. The recordings were conducted at a 1 kHz sampling rate with a maximum acceptable skin–electrode impedance of 5 kΩ. No digital filters were applied during the recording of the signal.

During the experiment, participants were instructed to perform right elbow flexion and extension movements or MI according to a visual stimulus presented on the screen. Each participant completed 60 executed movements and 60 MI trials. On the day before the experiment, participants received oral instructions regarding the experimental procedures and provided informed consent to participate in the study. Handedness was assessed using the Edinburgh Handedness Inventory [36], and motor imagery ability was evaluated using the Motor Imagery Questionnaire—Revised (MIQ-R) [37]. Additionally, each participant underwent a 10 min training session to become familiar with the experimental protocol and ensure proper habituation.

The NeuroSCP experimental protocol was explicitly designed to temporally segment flexion and extension phases, as well as distinct periods of attention, execution, and rest. As a result, the dataset includes five different classes: flexion movement, extension movement, flexion MI, extension MI, and rest. In this study, EEG data of flexion MI, extension MI, and rest from 12 NeuroSCP subjects were analyzed.

**Figure 2:**
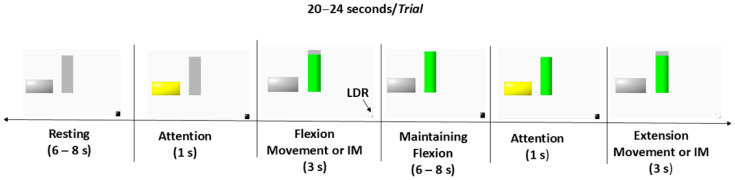
Schema representing the experimental paradigm of the NeuroSCP dataset. The horizontal rectangle turns yellow, representing a visual stimulus for attention. At the same time, the vertical green bar is a visual indication of the velocity at which the movement or IM should be executed.

The visual stimulus used in the experiment was developed using LabVIEW version 2022 Q3 software (National Instruments, Austin, TX, USA) and consists of a horizontal rectangle and a vertical bar displayed on the screen (Figure 2). After the attention period (Figure 2—indicated by a yellow rectangle), the vertical bar begins to fill from bottom to top in green, and the participant should perform an elbow flexion movement until reaching the maximum point, following the rate of filling of the vertical bar. This period corresponds to the flexion movement phase (Flex) and lasts for 3 s. After the bar is filled in green, the subject maintains the flexed position for 6–8 s. After this, the attention signal is presented again and remains on the screen for 1 s. Then, the green color of the horizontal bar is gradually replaced by gray from top to bottom, and the participant must perform an elbow extension movement until the forearm is entirely supported on the chair. This period corresponds to the extension movement phase (Ext) and also lasts for 3 s. After the extension movement, there is a resting period lasting for 6–8 s.

The beginning of the Flex and Ext periods is digitally marked by a synchronization signal, which utilizes a Light Dependent Resistor (LDR) sensor positioned at the bottom right corner of the screen. This enables precise temporal synchronization between the signals recorded by the acquisition system and the visual stimuli displayed on a monitor. This signal is triggered by a black square located in the lower right corner of the stimulus images (Figure 2), which turns white at the beginning of the movement period. This color change was detected by a photoelectric sensor placed on the monitor.

**Figure 3:**
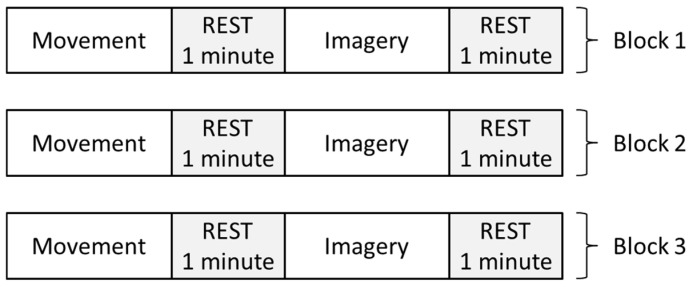
Schematic representation of block division of the NeuroSCP dataset. Each block takes up to 18 min (8 min for movement recording, 8 min for imagery recording, and 2 min for resting).

The experiment is divided into three blocks. Each block contains 20 movement sequences and 20 motor imagery sequences, with a 1 min pause between tasks (Figure 3) and a 1 min break between blocks.

### 2.3. Data Preprocessing

The same preprocessing steps were uniformly applied to both datasets. To assess whether variations in preprocessing parameters could influence the final classification outcomes, multiple filtering configurations were tested along with different time-window selections used as inputs for the feature extraction algorithms. Following acquisition, the signals were detrended and downsampled to 250 Hz to standardize the sampling frequency across both datasets. Additionally, a 60 Hz notch filter (Q-factor = 30) was used specifically for the NeuroSCP dataset, as the BCI dataset had already been notch-filtered. After this step, two different filters were applied to the EEG signals of both datasets: I—a 4th-order Butterworth band-pass filter between 4 and 45 Hz, and II—the same filter but with a band-pass set between 8 and 30 Hz. To better evaluate the classification performance under realistic conditions, we did not use artifact removal and rejection methods.

Since the NeuroSCP and BCI-C datasets differed in both the number and configuration of EEG channels, the FCz channel, which was present only in the BCI-C dataset, was excluded. Additionally, extra channels from the NeuroSCP dataset were removed to ensure that both datasets shared the same set of EEG channels for comparison. To assess whether increasing the number of input channels could improve classification accuracy, an additional analysis was conducted using all available EEG channels from the NeuroSCP dataset in the feature extraction process.

**Figure 4:**
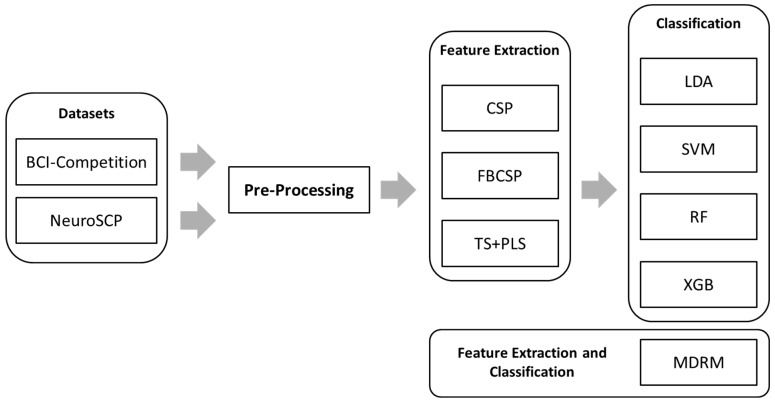
Data processing pipeline. After preprocessing, the data was used as input for three feature extraction techniques. Each feature was then classified using four classification algorithms. MDRM was also tested after preprocessing.

For this study, three datasets were analyzed: BCI-C, NeuroSCP 21 channels, and NeuroSCP 30 channels. Three different feature extraction methods were applied to each dataset: Common Spatial Patterns (CSP), Filter-Bank Common Spatial Patterns (FB-CSP), and Riemannian Tangent Space (TS) + Partial Least Squares (PLS). Four classification algorithms were used: Linear Discriminant Analysis (LDA), Support Vector Machines (SVM), Random Forest (RF), and Extreme Gradient Boosting (XGB). Additionally, the Minimum Distance to Riemannian Mean (MDRM), which is both a feature extraction and classification method, was tested. Each method was evaluated using four distinct time windows ([0–1 s], [0.5–1.5 s], [0–2 s], and [0.5–2.5 s], in which t = 0 s corresponds to the beginning of the IM period) to evaluate which combinations of temporal segments and feature extraction techniques would provide better separability between the different MI tasks. Altogether, this resulted in 96 different analyses. Figure 4 shows the data processing used in this study.

### 2.4. Feature Extraction Methods

#### 2.4.1. Common Spatial Patterns (CSP)

CSP is a valuable technique for extracting discriminative features from EEG data. It aims to define spatial filters that maximize the difference in EEG signal power between two or more classes. The CSP begins with the calculation of the covariance matrix for each class of EEG data, and then the covariance matrices are decomposed into their eigenvectors and eigenvalues. The eigenvectors corresponding to the largest and smallest eigenvalues are selected as the spatial filters, and the EEG data is projected onto these spatial filters, resulting in a new set of features where the variance between classes is maximized [18]. Two components were selected for the CSP implementation.

#### 2.4.2. Filter Bank Common Spatial Patterns (FB-CSP)

The FB-CSP technique comprises three stages: I—In the first stage, the EEG signals are bandpass-filtered into the frequency bands of interest. II—The CSP features are then extracted from each of these bands. III—A feature selection algorithm is used to select discriminative pairs of frequency bands and corresponding CSP features [21].

In this study, two combinations of frequency bands were used. For the band-pass filtered datasets (4–45 Hz), ten EEG bands were used: 4–8 Hz, 8–12 Hz, …, and 40–44 Hz. For the band-pass filtered datasets (8–30 Hz), five EEG bands were used: 8–12 Hz, 12–16 Hz, …, and 24–28 Hz. Four components were selected for each frequency band. Feature selection was performed based on mutual information criteria, and the top six pairs of components were selected for classification.

#### 2.4.3. Minimum Distance to Riemannian Mean (MDRM)

The MDRM classifier is a Riemannian geometry-based technique that operates on the covariance matrices of EEG signals, leveraging the geometric structure of the manifold of symmetric positive-definite (SPD) matrices. In this approach, each EEG trial is represented by its covariance matrix, which is treated as a point on the SPD manifold. Classification is then performed by computing the Riemannian distance between the trial covariance matrix and class means, assigning the new trial to the class with the minimum distance [38]. Covariance matrices were estimated using the Ledoit-Wolf shrinkage method [39].

#### 2.4.4. Riemannian Tangent Space (TS) + Partial Least Square (PLS)

The SPD matrices cannot be directly fed to vector-based classifiers because they assume that the data are distributed in a Euclidean space. The TS is a technique that projects each SPD matrix onto a tangent space, which is a locally flat space in which standard classifiers can be used. TS is a high-dimensional space with a dimensionality of N(N + 1)/2, where N is the number of channels, exceeding the number of MI EEG trials. For this reason, we used a feature reduction procedure. The Partial Least Squares (PLS) regression algorithm is a multivariate feature reduction method that explores the covariance between the predictor variables and target variables by finding a set of variables with maximal correlation [38].

### 2.5. Classification Methods

In this work, several classification algorithms were employed to discriminate between different MI tasks in both the BCI Competition dataset and the NeuroSCP dataset. Each classifier was evaluated across all possible combinations of time windows, feature extraction algorithms, and frequency filters. The classification methods included Linear Discriminant Analysis (LDA) [40], Support Vector Machines (SVM) [41], Random Forest (RF) [42], and Extreme Gradient Boosting (XGB) [43]. Additionally, the MDRM was applied, which serves as both a feature extraction and classification technique by operating directly on covariance matrices in the Riemannian framework. To implement this multiclass classification problem, a one-versus-rest strategy was employed. To evaluate the performance of the classification, a stratified five-fold cross-validation was applied.

The top components used in FB-CSP and the PLS components used in TS were estimated only in the training set. We tested up to 21 PLS components.

A grid search approach was used to optimize the hyperparameters of the SVM, RF, and XGB classifiers. For this approach, we used a stratified five-fold cross-validation only on the training set during each iteration, ensuring that parameter optimization did not use the test set.

For SVM, the following parameters were optimized: kernel function (linear, radial basis function), gamma γ (10^−5^ to 10), and regularization parameter c (10^−5^ to 10^2^). For RF classifier, was used the Gini impurity criterion and the following parameters were optimized: number of estimators (100, 200); maximum tree depth (None, 10, 20); minimum number of samples required to split an internal node (2, 5, 10); minimum number of samples needed to be at a leaf node (1, 2, 5); the number of features for the best split was selected according to either $\sqrt{p}$ or (*p*), where *p* is the number of features. The XGB classifier was evaluated using logarithmic loss, and the following parameters were optimized: number of boosting rounds (50, 100), maximum tree depth (3, 6), learning rate (0.01, 0.1, 0.3), subsample ratio of training instances (0.8, 1.0), subsample ratio of columns when constructing each tree (0.8, 1.0), and minimum loss reduction (0, 0.1, 0.2).

### 2.6. Transfer Learning Methods

To assess the limitations of cross-subject generalization, we tested a modified leave-one-subject-out (LOSO) strategy using the MDRM classifier. The training set included data from all subjects except the target subject. In addition, a subset of the target subject’s data was included in the training set using five-fold cross-validation, where the remaining folds were used as the test set. These results, together with the subject-specific results, provide a benchmark for evaluating the following two transfer learning methods for the NeuroSCP dataset.

The first method was the Minimum Distance to Weighted Means (MDWM), a Riemannian geometry-based extension of the MDM classifier designed for cross-subject transfer learning. This method extends the concept of composite mean from Euclidean space to Riemannian space by utilizing information from source subjects to enhance the classification of target subjects. For each class, the mean covariance matrices from all the source subjects are computed, and then combined with the mean covariance matrix of the target subject using an interpolation parameter λ [44,45].

The second method was Tangent Space Alignment (TSA). This Riemannian transfer learning algorithm operates in the tangent space by aligning the covariance matrices of the source subjects with the target domain using a Riemannian Procrustes Analysis approach. Covariance matrices from both source and target data are projected onto the tangent space at a standard reference by setting their global mean. In the tangent space, each subject’s data are centered, scaled, and rotated to align the mean of each class, thereby reducing the domain shift [46].

## 3. Results

### 3.1. Mean Classification Accuracy for Each Feature Extraction Technique

Table 2 presents the mean classification accuracies obtained using the CSP feature extraction method for both the BCI-C and NeuroSCP datasets. The highest mean accuracy for three-class motor imagery classification involving different limbs (BCI-C dataset) was 67.8% (F1-score 66.8%), achieved using the [0.5–2.5 s] time window, 8–30 Hz frequency range, and SVM classifier. For the NeuroSCP dataset with 21 channels, the best result was 48.6% (F1-score 47.2%), achieved using a time window of [0.5–2.5 s], 4–45 Hz frequency range, and an LDA classifier. When all 30 channels were used (NeuroSCP-30), the highest accuracy increased slightly to 51.5% with LDA and 51.6% with SVM (F1-score 49.3% and 49%) under the same temporal window. NeuroSCP classification accuracies are lower than those of BCI-C, and the inclusion of additional channels did not substantially improve classification performance.

**Figure 5:**
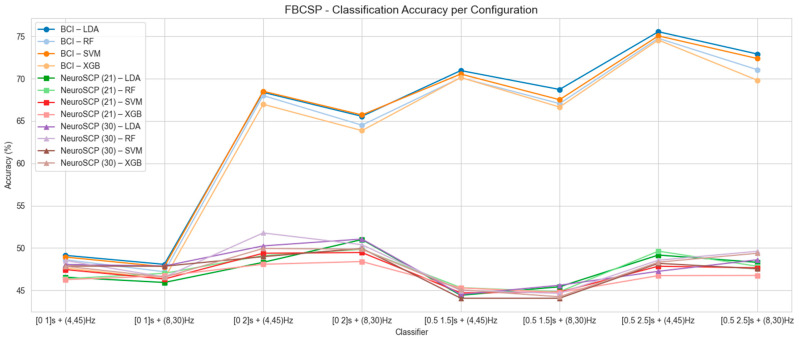
Mean classification accuracy using FB-CSP as a feature extraction technique for the BCI-C, NeuroSCP 21 channels (NeuroSCP 21), and NeuroSCP 30 channels (NeuroSCP 30) datasets.

**Figure 6:**
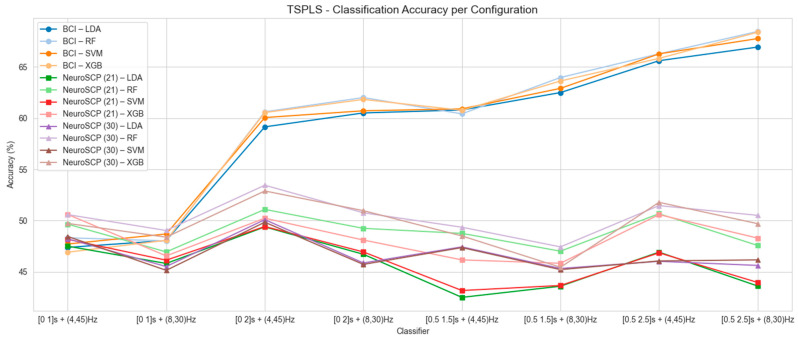
Mean classification accuracy using TS-PLS as a feature extraction technique for BCI-C, NeuroSCP 21 channels (NeuroSCP 21), and NeuroSCP 30 channels (NeuroSCP 30) datasets.

**Figure 7:**
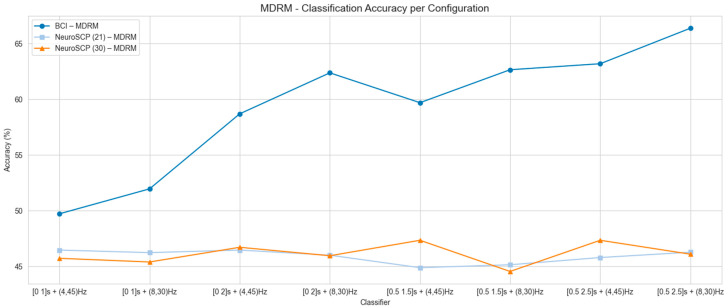
Mean classification accuracy using MDRM as a feature extraction technique for BCI-C, NeuroSCP 21 channels (NeuroSCP 21), and NeuroSCP 30 channels (NeuroSCP 30) datasets.

Figure 5, Figure 6 and Figure 7 present the mean classification accuracy results for all tested configurations, including time windows and frequency ranges, for the other feature extraction methods (FB-CSP—Figure 5, TS-PLS—Figure 6, and MDRM—Figure 7), as well as classification algorithms for both EEG datasets. The best classification accuracy results for the BCI-C dataset were achieved using the [0.5–2.5 s] time window, 4–45 Hz frequency range, FB-CSP for feature extraction, and LDA as the classifier (accuracy of 75.6% and F1-score 75.4%). The worst results for the BCI-C dataset were obtained when using a [0–1 s] time window. As for the NeuroSCP 30-channel dataset, the best results were obtained with FB-CSP and TS-PLS, [0–2 s] time window, 4–45 Hz frequency range, and RF as a classifier (53.4%, F1-score 53.2%). These results indicate that techniques commonly applied to classify different limb MI based on EEG features cannot perform well when classifying different MI tasks with the same limb.

Overall, the lowest accuracies were observed when using the [0–1 s] and [0.5–1.5 s] time windows in combination with the 8–30 Hz frequency range (Figure 5, Figure 6 and Figure 7). These results suggest that gamma (>30 Hz) and theta (4–8 Hz) frequency bands, which fall outside this range, play a significant role in distinguishing MI tasks involving the same limb. Based on these analyses, the configurations that produced the best mean classification performances were selected to assess subject-wise classification for the BCI-C and NeuroSCP subjects.

### 3.2. Subject-Wise Classification Accuracy Results

**Figure 8:**
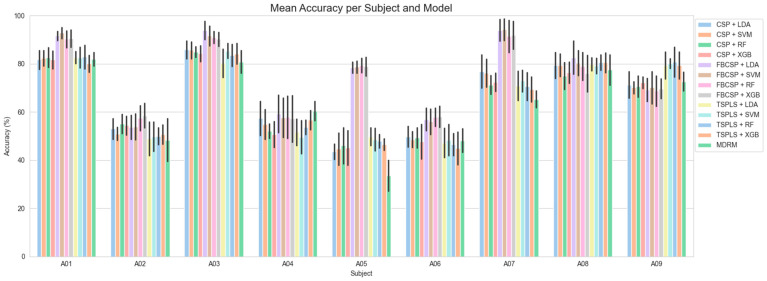
Subject-wise classification of the BCI-C dataset using [0.5–2.5 s] time window and 4–45 Hz frequency range with different feature extraction and classification algorithms.

**Figure 9:**
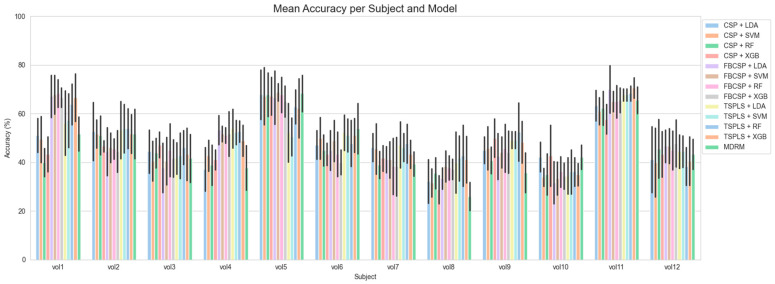
Subject-wise classification of the NeuroSCP using a [0–2 s] time window and a 4–45 Hz frequency range with different feature extraction and classification algorithms.

The subject-wise classifications of BCI-C and NeuroSCP are shown in Figure 8 and Figure 9, respectively. For BCI-C, the best subject classification accuracy results were achieved with 94% for subjects A3 and A7 (F1-scores of 94% for both), using FB-CSP feature extraction, LDA as the classifier, and a 4–45 Hz frequency band. The worst result using the same algorithms was 54% for subject A2. The time window that provided the best results was [0.5–2.5 s].

**Figure 10:**
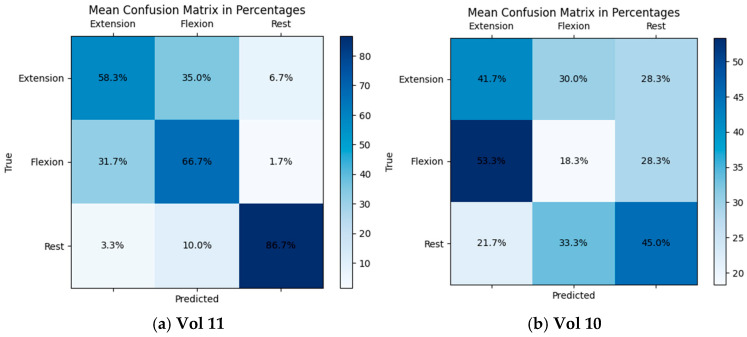
Mean confusion matrices for vol 11 and vol 10 of the NeuroSCP dataset using TS-PLS feature extraction, XGB as classifier, [0–2 s] time window, and 4–45 Hz frequency range.

Regarding NeuroSCP, as the inclusion of additional EEG channels did not substantially affect the classification performance, further analyses were conducted using only the 21-channel dataset. The best classification result achieved was 71% for the subject vol 11 (70.3% F1-score), using TS-PLS as feature extraction, XGB as the classifier, a time window of [0–2 s], and a 4–45 frequency range. The worst individual result using the same algorithms was 35% for subject vol 10 (33.3% F1-score), which is almost the chance level (33% for three classes). The confusion matrices for the best (vol 11) and worst (vol 10) individual classification results are shown in Figure 10. Under these specifications, only vol10 achieved a classification accuracy of below 40%. Six subjects had accuracies between 40% and 50%, two subjects between 50% and 60%, and two between 60% and 70%.

### 3.3. Transfer Learning for the NeuroSCP Dataset

As expected, our modified leave-one-subject-out (LOSO) strategy with the MDRM classifier often resulted in the worst results, except for one subject, vol 8, which already had the worst individual result for the subject-wise MDRM classification. One participant had a 30-point decline in performance. These poor results can be attributed to the significant intersubject variability.

Although two different transfer learning methods (MDWM and TSA) were tested on the 21-channel NeuroSCP dataset, no consistent improvement was observed in the subject-wise classification accuracy. In half of the subjects, the MDWM yielded better results, with an improvement of up to 6 points.

## 4. Discussion

In this work, different combinations of time windows, EEG frequency ranges, feature extraction, and classification algorithms are used to distinguish between MI tasks in two different datasets. The 21-channel BCI-C, which comprises the right and left hand and feet MI, and the 32-channel NeuroSCP dataset, comprising the right-arm flexion MI, right-arm extension MI, and resting classes. The same preprocessing steps were uniformly applied to the EEG signals from both datasets. The best mean classification result for BCI-C was 76%. When evaluating the subject-wise classification, the best-case accuracy was 94%, and the worst-case accuracy was 54%. Regarding the NeuroSCP dataset, the best average classification result was 53%. The best-case scenario for the subject-wise classification was 71%, and the worst case was 35%.

Therefore, both the mean and subject-wise classification results for NeuroSCP were lower than those obtained from BCI-C. This discrepancy may be attributed to the nature of the MI tasks that were evaluated. In BCI-C, classification was performed between the MI of distinct limbs (left hand, right hand, and feet), which typically elicits more spatially distinct cortical activation patterns. Methods based on covariance matrices (CSP and TS-PLS) can discriminate these spatially distinct patterns [38]. In contrast, NeuroSCP involved the distinction between different tasks involving the same limb (right arm flexion, right arm extension, and resting), which are more similar in their cortical representations and therefore more challenging to distinguish using EEG signals [29].

### 4.1. BCI-C Classification

The classification accuracies obtained in the present work for the BCI-C dataset were in line with those of other works using similar feature extraction and classifiers [47,48]. Some studies have used the 8–30 Hz [38,49,50] and 6–35 Hz [29,51] frequency ranges or even other frequency bands [16]. However, in this work, the best results were achieved using the 4–45 Hz frequency range with FB-CSP feature extraction. When applying CSP and TS-PLS, the difference in frequency ranges is not clear, and in MDRM, the 8–30 Hz range obtained a slightly better result. Therefore, the theta (4–8 Hz) EEG frequency band influenced the classification outcome for BCI-C in a feature extraction technique-dependent manner.

Other works have also used a time window starting at 0.5 s for EEG-MI signal feature selection [45,49]. The optimal time window length for promoting MI-BCI controlling classification accuracy without excessive delays was found to be 1–2 s [52]. Accordingly, in this work, the best results were obtained with a 2-s length time window. Our results suggest that for the BCI-C dataset, feature extraction using a time window starting 0.5 s after stimulus onset provides better results than starting at 0 s, regardless of the feature extraction technique used. This hypothesis is based on the fact that the BCI-C experimental protocol includes an auditory stimulus in addition to a visual stimulus at t = 0 s. The robust visual and auditory potentials elicited by these stimuli comprise components that can last up to 400 ms [53] and can mask the early cognitive task signals of the MI.

When evaluating the subject-wise classification accuracy results, we can confirm previous findings that show far greater accuracies for some subjects than for others [48]. The analysis involving the BCI-C dataset was used to compare the results obtained with NeuroSCP.

### 4.2. NeuroSCP Classification

This work proposes an evaluation of the classification performance under close-to-realistic conditions. Therefore, no artifact removal or rejection methods were applied. *A posteriori* analysis of the subjects with the worst NeuroSCP classification accuracy revealed high contamination of EEG signals with artifacts. The presence of artifacts causes wave distortions that can affect pattern recognition [54]. Therefore, the significant variability in the subject-wise classification results obtained could be due to contamination of the EEG signals with EMG, EOG, or artifacts from other sources, which reduced the mean classification accuracy.

The inclusion of the additional EEG channels recorded in the NeuroSCP dataset did not substantially affect classification performance. The time window that produced the best mean classification accuracy was [0–2 s]. The difference in optimal time windows between the two experimental protocols may be attributed to the reduced presence of visual-evoked potentials in the NeuroSCP dataset, as the vertical and horizontal bars remained constantly visible on the screen in front of the subjects. Additionally, no auditory evoked potentials were expected since the experiments were conducted in a silent room, and no auditory stimuli were presented to the participants.

The low classification accuracies observed in the NeuroSCP dataset may reflect the limited suitability of current feature extraction techniques for distinguishing between different MI tasks involving the same limb. As a perspective for future research, we suggest exploring alternative approaches, such as EEG functional connectivity measures, which may better capture the distributed cortical dynamics underlying subtle differences in tasks.

Functional connectivity methods derived from Granger causality, including Partial Directed Coherence and the Directed Transfer Function [55], can capture directed interregional interactions during motor imagery tasks, which may reveal subtle differences in the same-limb- MI that spectral features alone cannot detect. These measures have been successfully used to improve MI classification performance [56] and to characterize functional reorganization in motor networks during rehabilitation [57]. Another possible direction involves the application of movement intention detection algorithms, which may enhance classification performance by detecting motor planning-related signals that are not evident in conventional spectral features.

Other works have also applied TS-PLS feature extraction, followed by LDA and SVM for classification. They reported accuracies of around 80.5% for the 6-class MI tasks of the same limb [49]. However, the experimental protocol adopted is substantially different from the one proposed in this work, as the subjects had to perform the instructed MI task multiple times per trial. Another difference lies in the sliding time window used for EEG trial segmentation, where each time window overlaps with the previous one by 80%. This could have influenced the classification accuracy results, as only 20% of the samples differed from the previous one for each time window. Furthermore, the authors used Independent Component Analysis (ICA) to remove EMG and EOG artifacts from EEG data, rejecting 10 independent components. ICA is an important tool for rejecting artifacts from EEG signals; however, it may not be readily applied in an online BCI system context due to its high computational cost and time consumption. These methodological differences may explain the differences in the results for the same limb MI tasks.

## 5. Conclusions

In conclusion, our findings suggest that the feature extraction algorithms used to distinguish between MI with different limbs are not effective for different MI tasks involving the same limb. We propose other techniques, such as EEG functional connectivity or a hybrid approach combining a movement intention detection algorithm with classification techniques, to be tested in future work.

## Figures and Tables

**Table 1:** Recent studies addressing the classification of different MI tasks involving the same limb and MI tasks involving different limbs, including feature extraction methods, classification algorithms, number of subjects (volunteers), task classes, classification accuracies, and standard deviations in parentheses.

Year	Title	Feature Extraction	Classifiers	N Vol	Classes	Accuracy
2015	EEG Classification of Different Imaginary Movements within the Same Limb [29]	BP, CSP, FB-CSP	LDA, LR, SVM	12	3-Classes: Rest, Grasp, Elbow	56.2% (8.5)
					3-Classes: Rest, Grasp, Elbow (on Goal)	60.7% (8.4)
2019	Deep Channel-Correlation Network for Motor Imagery Decoding From the Same Limb [30]	Correlation, MSC	Channel Correlation CNN	25	3-Classes: Rest, Hand, Elbow	87%
2020	Decoding Hand Motor Imagery Tasks Within the Same Limb from EEG Signals Using Deep Learning [27]	CNN	CNN	20	2-Classes: Flexion, Extension	78.46% (12.5%)
					3-Classes: Flexion, Extension, Grasping	76.7% (11.7%)
2021	Discriminating Three Motor Imagery States of the Same Joint for Brain–Computer Interface [31]	TDP, CSP, FB-CSP, EMD-CSP, LMD-CSP	LDA, ELM, KNN, SVM, LS-SVM, MOGWO-TWSVM	7	3-Classes: Abduction, Flexion, Extension of the shoulder	91.6%
2021	EEG-Inception: An Accurate and Robust End-to-End Neural Network for EEG-based Motor Imagery Classification [33]	CNN	CNN	9	BCI-C IV-2a Right and left hands, both Feet, and Tongue	88.4% (7)
				9	BCI-C IV 2b Right and left hands	88.6% (5.5)
2023	Recognition of Motor Intentions from EEGs of the Same Upper Limb by Signal Traceability and Riemannian Geometry Features [32]	FB-CSP, Riemannian geometry	SVM	15	6-Classes: Grasping and holding of the palm, Flexion and Extension of the elbow, Abduction/Adduction of the shoulder	22.5% (3)

**Table 2:** Classification accuracy results, means, and standard deviation, using CSP feature extraction and different frequencies, time windows, and classification algorithms for the BCI Competition dataset. The numbers in bold represent the best results for each dataset.

		4–45 Hz				8–30 Hz			
Dataset	Classifier	[0 1]	[0 2]	[0.5 1.5]	[0.5 2.5]	[0 1]	[0 2]	[0.5 1.5]	[0.5 2.5]
BCI-C	LDA	46.51 (8.95)	58.29 (10.69)	62.99 (14.01)	66.54 (15.72)	49.92 (7.41)	60.39 (11.75)	63.71 (15.78)	67.44 (16.69)
	RF	45.29 (8.45)	57.95 (11.06)	60.42 (12.30)	65.23 (14.79)	47.43 (8.66)	58.82 (12.44)	62.65 (14.92)	66.05 (17.12)
	SVM	46.89 (8.75)	59.31 (10.75)	62.78 (13.26)	65.88 (16.06)	49.71 (7.55)	60.70 (11.50)	63.86 (15.75)	**67.83 (15.82)**
	XGB	45.24 (8.35)	57.41 (11.53)	59.52 (13.66)	64.99 (15.41)	48.17 (8.44)	58.15 (12.81)	61.39 (15.83)	65.95 (15.61)
NeuroSCP (21 channels)	LDA	47.25 (11.96)	47.55 (10.13)	47.20 (9.09)	**48.61 (9.17)**	46.06 (12.01)	47.08 (10.62)	47.11 (9.76)	47.08 (9.74)
	RF	44.84 (9.00)	45.39 (10.26)	45.60 (8.70)	45.97 (7.47)	45.37 (10.76)	47.06 (9.91)	45.16 (9.57)	43.82 (8.76)
	SVM	46.41 (11.67)	46.64 (10.28)	46.34 (9.93)	48.17 (9.42)	45.46 (11.38)	46.09 (10.64)	46.46 (9.80)	44.56 (10.95)
	XGB	44.44 (10.69)	46.11 (9.41)	44.81 (8.10)	45.58 (7.44)	45.19 (9.54)	45.83 (9.59)	43.98 (9.50)	43.61 (9.32)
NeuroSCP (30 channels)	LDA	47.13 (9.30)	50.51 (9.24)	49.54 (7.26)	**51.53 (8.35)**	47.92 (10.81)	50.35 (9.50)	46.06 (7.43)	49.68 (8.25)
	RF	47.25 (9.91)	49.40 (9.59)	46.55 (6.05)	51.16 (10.79)	45.97 (9.72)	49.24 (8.06)	45.28 (7.70)	48.29 (7.89)
	SVM	47.64 (9.79)	49.63 (10.36)	47.96 (7.01)	**51.69 (9.29)**	47.20 (8.78)	49.54 (9.34)	46.41 (8.11)	49.12 (8.21)
	XGB	44.98 (9.94)	47.92 (9.03)	47.57 (5.71)	50.69 (8.70)	45.88 (9.07)	49.21 (7.04)	45.02 (7.85)	48.56 (8.66)

## Data Availability

The datasets presented in this article are not available because the Ethics Committee that approved our protocol did not authorize the sharing of raw EEG data with individuals or institutions outside the original scope of the study. Therefore, public sharing of the datasets is not permitted under the terms of the approved ethical guidelines.
